# Rapidly progressive primitive neuroectodermal tumor of the prostate: A case report and review of the literature

**DOI:** 10.3892/ol.2014.2731

**Published:** 2014-11-24

**Authors:** TADAMASA SHIBUYA, KENICHI MORI, YASUHIRO SUMINO, FUMINORI SATO, HIROMITSU MIMATA

**Affiliations:** Department of Urology, Faculty of Medicine, Oita University, Yufu, Oita 879-5593, Japan

**Keywords:** primitive neuroectodermal tumor, prostate, rapid progression

## Abstract

The present study reports a rare case of primitive neuroectodermal tumor (PNET) of the prostate. A 23-year-old male presented to Oita Medical Center (Oita, Japan) with the complaint of dysuria and anal pain. A large mass in the prostate and a number of swollen lymph nodes in the pelvic region were identified by a computed tomography scan and magnetic resonance imaging. The patient was, thus, admitted to Oita University Hospital (Yufu, Japan), where a biopsy of the prostate gland was performed. Histological analysis revealed small round cells that were positive for MIC-2 expression and fluorescent *in situ* hybridization analysis detected a translocation involving Ewing sarcoma breakpoint region 1 at chromosome 22q12. Thus, a diagnosis of PNET of the prostate was established. Systemic chemotherapy was the selected treatment, however, a poor response was obtained. The patient succumbed approximately four months after the initial onset of symptoms. PNET of the prostate has been reported in eight cases worldwide; in comparison, the present case exhibited the most unsatisfactory outcome.

## Introduction

Primitive neuroectodermal tumor (PNET) is included in the Ewing family of tumors (ESFTs) as they exhibit identical cytogenetic changes. The specific translocation t(11;22)(q24;q12) was identified in Ewing’s sarcoma and PNET, however, various other translocation patterns may also be involved (1). ESFTs, which also include extraosseous Ewing’s sarcoma and Askin’s tumor (Ewing’s sarcoma of the chest wall) are aggressive malignant primary tumors of the bone, which are the second most common bone tumor in children and adolescents. PNET is divided into central PNET and peripheral PNET according to their location or origin. There are various symptoms in peripheral PNET which can occur in almost all areas of the body except for the central nerve system (2,3). A common feature of image findings of peripheral PNET is a large and infiltrative soft tissue mass with an ill-defined, necrotic region and heterogeneous enhancement (2,4). However, it is not easy to diagnose peripheral PNET by imaging alone due to insufficient radiological studies (2). Combination therapy including chemotherapy, radiation therapy and surgery are chosen for treatment against peripheral PNET (5), however the patient prognosis is poor (6). Recently, a few cases of PNET occurring in the urologic region such as bladder, kidney and adrenal glands have been described (7,8). PNET of the prostate, which was also classified as peripheral PNET, was first reported in 2003 (9). To the best of our knowledge, the present study reports the ninth case of PNET of the prostate gland thus far. Written informed consent was obtained from the family of the patient.

## Case report

**Figure 1:**
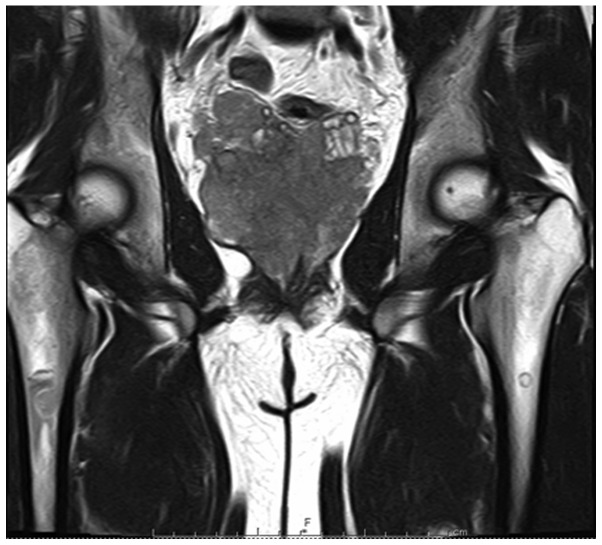
Coronal T2-weighted magnetic resonance image demonstrating a large tumor in the prostatic legion.

**Figure 2:**
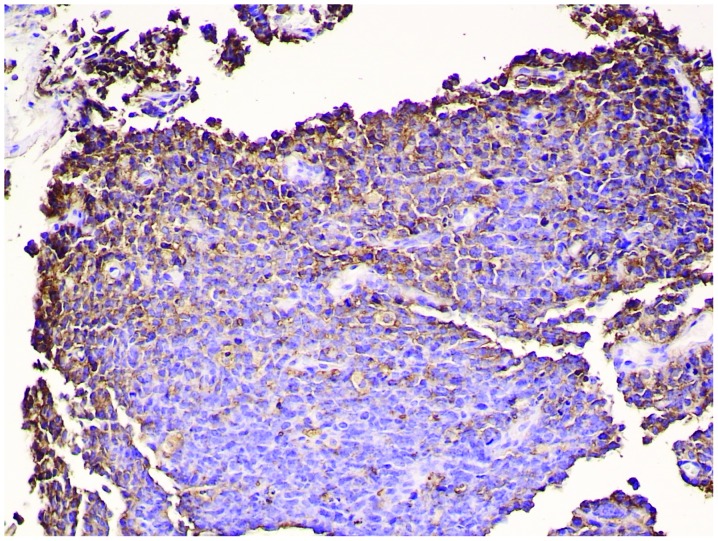
Immunohistochemical staining revealing small round cells positive for MIC-2. Magnification, ×400.

**Figure 3:**
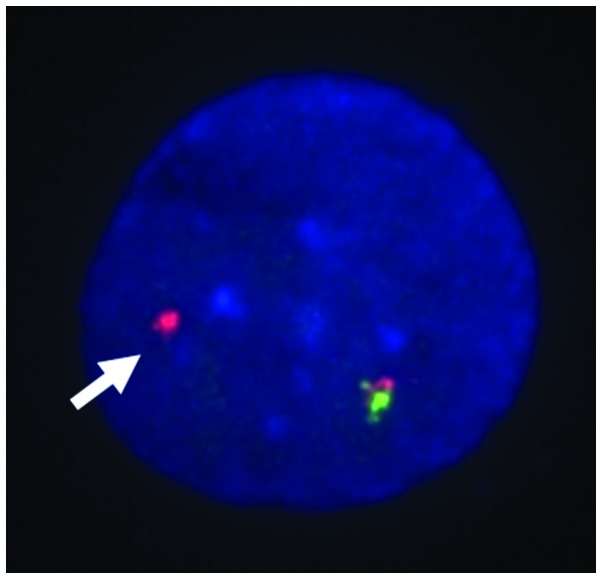
Fluorescence in situ hybridization analysis demonstrated split signals (arrow) using a Ewing sarcoma breakpoint region 1 probe at chromosome 22q12. A split signal was observed in 99/100 cells. Magnification, ×1000.

A 23-year-old male presented to Oita Medical Center (Oita, Japan) with the complaint of dysuria and anal pain. A computed tomography (CT) scan and magnetic resonance imaging (MRI) revealed a large solid tumor in the pelvic region replacing the prostate (Fig. 1) and multiple swollen lymph nodes. Cystostomy and biopsy of the prostate gland were performed. Histopathological analysis demonstrated small round tumor cells, indicating a PNET, however, no rosette structure was observed. The tumor cells were positive for MIC-2 (Fig. 2), cytokeratin, vimentin and neural cell adhesion molecule, but negative for the other immunostains, chromogranin A, neurofilament, neuron-specific enolase, leukocyte common antigen, desmin, HHF35, sarcomeric actin, myogenic differentiation 1 and myoglobin. The histopathological results indicated a diagnosis of PNET of the prostate, and the patient was admitted to Oita University Hospital (Yufu, Japan) for treatment. Two days after admission, the patient complained of lower back pain. Re-examination by CT scan and MRI revealed multiple bone metastases to the dorsal and lumbar vertebrae, pelvic bone and bilateral thighbones, multiple lung metastases and a fracture of the fourth lumbar vertebra caused by metastasis. An additional genetic examination was performed; the Ewing’s sarcoma-friend leukemia virus integration 1 fusion gene was not detected by quantitative polymerase chain reaction, however, split signals were observed in fluorescence *in situ* hybridization (FISH) analysis using a Ewing sarcoma breakpoint region 1 (EWSR1) probe (SRL Laboratories, Inc., Tokyo, Japan; Fig. 3). A diagnosis of PNET of the prostate was established, and treatment with systemic chemotherapy commenced. Following one cycle of chemotherapy (ifosfamide, 2 mg/m^2^/week) the patient exhibited various side-effects, including dizziness, headache and nausea. MRI revealed multiple metastases to the intracranial meninges and a CT scan indicated a poor chemotherapy response. Therefore, best supportive care was administered and the patient succumbed approximately four months after the initial onset of symptoms.

## Discussion

A multimodal therapeutic regimen that includes a combination of chemotherapy, surgery and radiotherapy is the current treatment strategy for the Ewing sarcoma family of tumors. Additionally, PNET of the prostate is commonly treated by combination therapy (Table I) (9,12–18). In all but one of the reported cases of PNET of the prostate, in which the treatment strategy was not detailed, neoadjuvant or adjuvant chemotherapy were effectively administered. Two cases in the literature exhibited a reduction in the size of the primary tumor following neoadjuvant chemotherapy (12,15) and four cases underwent radical prostatectomy (9,12,15,18). Chemotherapeutic agents, including ifosfamide, etoposide, vincristine, Adriamycin and doxorubicin, were used as single or combination therapies. Although a standard treatment has not yet been established, chemotherapy is a potentially effective treatment for localized PNET of the prostate (1). In the present study, however, multiple metastases in various regions inhibited the effectiveness of the chemotherapy. Previously, the presence of metastatic disease was reported as the most unfavorable prognostic factor for Ewing sarcoma family tumors (19). In particular, patients exhibiting extrapulmonary metastatic disease have demonstrated a worse prognosis compared with individuals exhibiting solitary pulmonary metastatic disease. Furthermore, tumor size (>100 ml), location within the pelvis, older age (>14 years) and insufficient response to initial therapy are reported as poor prognostic factors (20–22). In the current study, the presence of a number of these poor prognostic factors resulted in unsatisfactory progress. Thus, treatment for high-risk patients may require the development of a novel therapeutic regimen distinct from those used to treat local cases of Ewing’s sarcoma, for example gemcitabine, docetaxel or targeted molecular agents.

The present case lacked the specific Ewing’s sarcoma/PNET translocation t(11;22)(q24;q12), however, EWSR1 gene rearrangement on chromosome 22 was detected by performing FISH analysis. Overall, ~15% of histopathologically defined MIC-2-positive Ewing sarcomas lack the classical specific translocation. However, in the majority of the remaining cases, variant translocations involve chromosome 22q12. Chromosome 21q22 (10% of Ewing sarcoma), and chromosomes 7q22, 17q12 and 2q36 (<1% of Ewing sarcoma) were reported as translocation partners (5), however, no translocation partner could be detected in the present case. Identifying the translocation partner may be important as the translocation type may contribute to an unfavorable prognostic outcome.

In all reported cases of PNET of the prostate gland, a large-sized primary tumor replaced the prostate at the diagnosis. Despite the large size of the tumor in the pelvic area, the predominant complaint of the patients was the common symptom of dysuria. Thus, PNET of the prostate gland should be considered when young males (20–30 years old) present to hospital with the complaint of dysuria, to improve the rate of early diagnosis. Non-invasive examinations, including ultrasound or digital rectal examination, are sufficient to identify the presence of this disease in the prostatic region.

## Figures and Tables

**Table I:** Clinical characteristics of reported primitive neuroectodermal tumor of prostate cases.

Case, n	First author (reference)	Age, years	Metastases	Chemotherapeutic agents	Radiation	Surgery	Survival, months
1	Colecchia *et al* (9)	31	None	Ifosfamide, etoposide, vincristine, Adriamycin	Yes	Yes	N/A
2	Peyromaure *et al* (12)	27	None	Ifosfamide, etoposide, vincristine, doxorubicin	Yes	Yes	2
3	Thete *et al* (13)	26	None	N/A	N/A	N/A	N/A
4	Kumar *et al* (14)	25	None	Ifosfamide, vincristine, actinomycin D, Adriamycin	N/A	N/A	N/A
5	Funahashi *et al* (15)	20	Lung	Ifosfamidenone	No	Yes	10
6	Mohsin *et al* (16)	29	Lymph node Lung	First-line; vincristine, doxorubicin Second-line; docetaxel, gemzar Third-line; ifosfamide	No	No	N/A
7	Al Haddabi *et al* (17)	24	None	Ifosfamide, doxorubiin, vincristine, etoposide	No	No	N/A
8	Wu *et al* (18)	29	Lung	Multiagent (detail N/A)	No	Yes	12

N/A, not available.
